# Risk of seroconversion and seroreversion of antibodies to *Chlamydia trachomatis* pgp3 in a longitudinal cohort of children in a low trachoma prevalence district in Tanzania

**DOI:** 10.1371/journal.pntd.0010629

**Published:** 2022-07-13

**Authors:** Xinyi Chen, Beatriz Munoz, Harran Mkocha, Charlotte A. Gaydos, Laura Dize, Thomas C. Quinn, Sheila K. West

**Affiliations:** 1 Dana Center for Preventive Ophthalmology, Wilmer Eye Institute, Johns Hopkins School of Medicine, Baltimore, Maryland, United States of America; 2 Kongwa Trachoma Project, Kongwa, Tanzania; 3 International Chlamydia Laboratory, Division of Infectious Diseases, Johns Hopkins School of Medicine, Baltimore, Maryland, United States of America; 4 Division of Intramural Research, National Institutes of Allergy and Infectious Diseases, National Institute of Health, Bethesda, Maryland, United States of America; University of Kansas School of Pharmacy, UNITED STATES

## Abstract

**Background:**

Serologic testing for chlamydial antibodies is one potential tool for trachoma monitoring. Understanding the dynamics of seroconversion and seroreversion in low endemic districts is critical for determining the value of using serology.

**Methodology/Principal findings:**

We surveyed a random sample of 2536 children aged 1–9 years in Kongwa, Tanzania, over three years; 1719 (67.8%) participants had all three follow-ups. Surveys assessed trachomatous inflammation—follicular (TF), *Chlamydia trachomatis* infection, and anti-pgp3 antibodies. Mass drug administration occurred immediately after the first and second follow-up surveys. The cohort was classified into trajectories of change in serostatus, and risk factors were evaluated for seroconversion and seroreversion. We found that 86.2% of seropositives remained seropositive throughout the study, whereas 12.1% seroreverted. Seroreverters were younger (Odds Ratio [OR] = 0.88 for every one-year increase in age, 95% CI = 0.79–0.99). 84.5% of seronegatives remained seronegative, and 13.0% seroconverted. Seroconverters were also younger (OR = 0.92, 95% CI = 0.87–0.98). Seroconversion and seroreversion were not explained by indeterminate values for the intensity of antibody response. Less than 1% of the cohort had unstable changes in serostatus, mostly explained by values in the indeterminate range. TF and infection in the cohort declined over time, while seropositivity increased from 31.5% to 36.4%.

**Conclusions/Significance:**

Antibody status is relatively stable over time. Both seroconversion and seroreversion occurred over the three years in this low endemic district, especially in younger children. Modeling seroreversion is important for accurate determination of seroconversion. The use of serology as a monitoring tool should target the younger aged children as they will most likely capture recent changes in serostatus.

## Author summary

Trachoma, the leading infectious cause of blindness worldwide, is a chronic conjunctivitis caused by repeated infection with *Chlamydia trachomatis*. As more endemic regions approach the trachoma elimination goal, it becomes critical to identify tools that can be used for monitoring the potential resurgence or re-emergence of trachoma. Serologic testing for chlamydial antibodies is one potential tool. Understanding the risk of seroconversion and seroreversion in children in low endemic districts is critical for determining the value of serology as a monitoring tool. We followed a cohort of children in the formerly trachoma endemic Kongwa district in Tanzania over three years. We demonstrated the relative stability of antibody status with 85–86% of the children retaining their baseline serostatus. We also documented an overall seroconversion rate of 13% and a seroreversion rate of 12%. We found younger ages to be a risk factor for both seroconversion and seroreversion. Our study provides necessary new evidence for the slow rate of overall change in seroprevalence over three years and highlights the potential use of the younger aged children for sero-monitoring of trachoma as they are more likely to reflect the dynamic interplay of true seroconversion and seroreversion. Furthermore, using only cross-sectional serostatus to estimate seroconversion may result in an underestimate if seroreversion rates are not included in the estimation.

## Introduction

Trachoma, the leading infectious cause of blindness worldwide, is a chronic conjunctivitis caused by repeated infection with *Chlamydia trachomatis* (Ct) [1]. The World Health Organization (WHO) set one of the elimination goals as sustained district-level prevalence of trachomatous inflammation–follicular (TF) in children aged 1–9 years less than 5% [2]. This is measured in two surveys at least two years apart with no intervening Mass Drug Administration (MDA). As more endemic regions approach the elimination goal, it becomes critical to identify proper methods of detecting re-emergence. If surveys find that trachoma increases (or goes above 5% in the case of trachoma surveillance surveys), more resource may need to be mobilized to manage resurgence.

However, the precision of TF grading becomes problematic when trachoma prevalence is low. There is tendency to overcall follicles [3], and there is clearly intergrader variability even when agreement is acceptable [4]. At the same time, a test of infection for surveillance purposes is also controversial as sensitive commercial tests are expensive, there are no guidelines or standardization on usage, and evidence shows that a low level of infection does not necessarily lead to trachoma re-emergence [5,6].

Serologic testing for chlamydial pgp3 antigen is a promising tool. Compared to surveys of TF and infection, which are snapshots of the current prevalence, early data suggest that seropositivity measures cumulative exposure to ocular Ct [7]. In areas where TF remains hyperendemic, antibody prevalence is high with increases in seropositivity with age, indicating ongoing transmission [7,8]. In surveillance surveys where TF was <5%, with low or almost absent infection, seropositivity rates were low, with zero to modest rates of increase with age [3,9,10]. A recent study identified re-emergence of trachoma based on TF of 7.1%, verified by infection and photo confirmation of clinical grades. There was a seroprevalence of 18.2% that increased with age; notably, a seropositivity rate of 6.7% in 1-2-year-olds, who were born after cessation of MDA, was strong evidence for ongoing transmission [11].

While serologic data provide useful information, there is limited understanding of the long-term stability of seropositivity in children in formerly endemic districts. To fully model district rates of seroconversion and possible transmission, this understanding is critical. A small study in a trachoma hyperendemic village found that seropositivity in a cohort of children was stable over six months. While MDA lowered the Median Fluorescence Intensity value of the antibodies, no child lost seropositivity [7]. However, in a district with TF of 5.2%, we followed a cohort of children in the absence of MDA over a year and found 6.4% of seropositive children at baseline seroreverted at one year; seroreverters were also more likely to live in communities with TF<5% [12]. Six months after one round of MDA, the seroreversion rate was 4% (estimated yearly rate of 8%) [13]. However, the stability of these changes over many years is unknown.

In this study, we followed a cohort of children over three years in a low endemic district and determined the stability of their antibody status and trajectories of change in serostatus. We hypothesized that seroconversion and seroreversion were more common in younger children, and that children residing in communities with TF≥5% were more likely to seroconvert while children residing in communities with TF<5% were more likely to serorevert.

## Methods

### Ethics statement

Written consent was obtained from the guardians of the children, and verbal assent was also obtained for participants aged ≥7 years. This study was approved by the Institutional Review Board of the Johns Hopkins School of Medicine and Tanzania National Institute for Medical Research.

### Study design

We randomly selected 52 of 90 communities in Kongwa, Tanzania [14]. Two of the 52 communities opted out of the study. A study census was carried out in the communities. Between August 2014 and February 2015, 2536 participants aged 1–9 years were randomly selected for the baseline survey, with an average of 51±4 randomly selected participants from each community (range: 41–60). There was no MDA between the baseline survey and the first follow-up. The first follow-up was conducted from February to April 2016, with MDA for all residents in the district at the end of April 2016. The second follow-up was conducted between October and December 2016, prior to another MDA in December. The third follow-up was performed from October 2017 to January 2018, approximately one year after MDA. The baseline and follow-up surveys assessed clinical trachoma, Ct infection, and antibodies to Ct antigen pgp3, discussed further below. The MDAs were provided by the National Trachoma Control Programme of Tanzania and supplemented for children aged 1–9 years by the Kongwa Trachoma Project. Estimated coverage in the cohort was over 90%.

### Surveys

One grader, standardized by a Global-Trachoma-Mapping-Project-trained grader, examined both eyes of each participant for signs of TF and trachomatous inflammation-intense (TI) using the simplified WHO criteria [15].

A swab was taken of the left tarsal conjunctiva of every child, stored dry in a refrigerator for up to 30 days, and shipped to Johns Hopkins University, where it was stored at -80°C until processed. A strict protocol was implemented to avoid field contamination. At least two field air controls were taken in every community by passing a sterile swab within 1 inch of the conjunctiva of a child from a randomly selected 5% sample. The laboratory personnel were masked to field controls versus ocular samples. None of the field controls were positive, indicating an absence of contamination.

Finger prick blood was collected from each participant and deposited onto six circular extensions on a filter paper calibrated to hold 10 μl each. The blood spots were dried and stored at -20°C until shipped to the International Chlamydia Laboratory at Johns Hopkins University, where they were processed within 90 days for antibodies to pgp3 on Luminex 100 platform.

### Laboratory processing

The ocular swabs were processed using the APTIMA Combo 2 (Hologic Inc., San Diego CA) commercial test with a pooling strategy with 4–5 samples per pool [16]. For pools that yielded a negative result, all specimens in that pool were considered negative for Ct. For pools that yielded a positive result, the original individual samples were retested to determine which sample(s) was positive for Ct. Equivocal/invalid pools were retested and, if still invalid, deconstructed for individual sample analyses. There were no invalid tests on the individual samples.

The processing of the dried blood spots was detailed elsewhere [17]. Briefly, serum eluates from the blood spots were incubated with pgp3-coupled beads. Biotinylated mouse anti-human total IgG (clone H2; Southern Biotech, Birmingham, AL), IgG4 (clone HP6025; Invitrogen, South San Francisco, CA), and R-phycoerythrin-labeled streptavidin (Invitrogen, South San Francisco, CA) were added. After washing, beads were read on a BioPlex 100 instrument (Bio-Rad, Hercules, CA) using Bio-Plex Manager 6.0 software (Bio-Rad). The level of fluorescence from each sample was reported as the Median Fluorescence Intensity Minus Background Intensity (MFI-BG). The background was the intensity of beads with only buffer. The cutoff for antibody positivity was determined by receiver operative characteristic (ROC) curve analyses, using sera from children who were positive for both infection and clinical trachoma and a sample of US children who had never been exposed to trachoma [17]. The indeterminate range was defined as the interval between the highest MFI-BG from the negative panel and the lowest MFI-BG from the positive panel. Two different bead sets were used: one for baseline and one for all the follow-up surveys. The two cut-offs for seropositivity were: 575 (log MFI-BG = 2.76) at baseline with an indeterminate range of 466–683, and 989 (log MFI-BG = 2.99) at follow-ups with an indeterminate range of 791–1187.

### Data analyses

All data were analyzed using R version 4.0.0. We divided the children with complete follow-ups (the cohort) into groups according to trajectories of change in serostatus. Continuous variables were compared using Wilcoxon rank-sum tests. Categorical variables were compared using χ2 tests. The calculation of cross-sectional TF, infection, and seropositivity proportions used all the participants that showed up at the specific survey; potential changes in proportions over time were evaluated for statistical significance using general estimating equations, accounting for serial correlation within children. For the cohort analyses, logistic regression was used to determine the contribution of age, sex, and community baseline TF to the probability of seroreversion (belonging to the seroreverter group versus the group who was seropositive throughout) or seroconversion (belonging to the seroconverter group versus the group who was seronegative throughout). A generalized estimating equation was used to account for clustering at the village level. We also conducted sensitivity analyses by including children who were lost to only the first or second follow up but whose serostatus could be determined from existing follow-ups.

## Results

**Fig 1:**
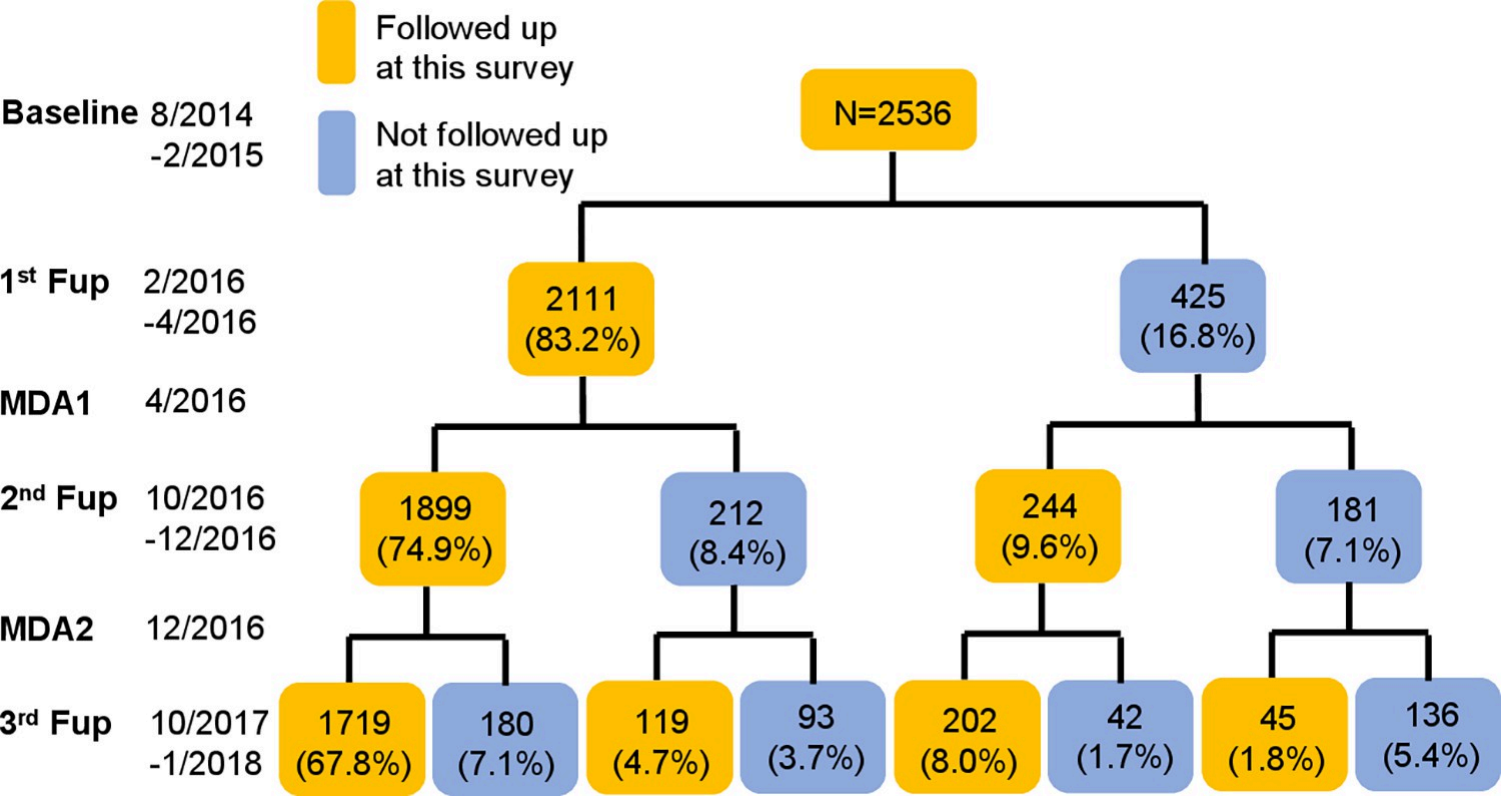
Flow chart of study participants. The denominator of the percentages is N = 2536. Follow up is denoted as Fup.

A total of 2536 children were surveyed at baseline and 1719 (67.8%) showed up to all four surveys (Fig 1). The cohort consisted of these 1719 children.

The percent of females was greater in the cohort compared to those who missed one follow-up (52.2% vs. 44.3%, P = 0.0023, Table 1). There was no difference by age, baseline TF, infection, or seropositivity.

**Table 1:** Baseline characteristics of the cohort who completed all follow-up visits versus those who missed one or more follow-ups.

Baseline characteristic	Longitudinal cohort (n = 1719)	Lost to one follow-up (n = 501)	*P* value^c^	Lost to two or three follow-ups (n = 316)	*P* value^c^
Age in years, mean (SD)	5.3 (2.5)	5.5 (2.5)	0.10	5.4 (2.6)	0.30
Age > 6 years, n (%)	610 (35.5)	199 (39.7)	0.093	123 (39.0)	0.25
Female, n (%)	897 (52.2)	222 (44.3)	0.0023	161 (51.1)	0.77
TF^a^, n (%)	94 (5.5)	21 (4.2)	0.31	17 (5.4)	1.0
TI^b^, n (%)	23 (1.3)	6 (1.2)	0.98	3 (1.0)	0.77
Infection, n (%)	78 (4.5)	32 (6.4)	0.12	15 (4.8)	0.98
Antibody positive, n (%)	522 (30.4)	158 (31.5)	0.66	120 (38.1)	0.0081

^a^ Trachomatous inflammation—follicular

^b^ Trachomatous inflammation—intense

^c^ Compared to the cohort (n = 1719)

The cross-sectional TF, infection, and seropositivity proportions of all the participants that showed up to each survey are shown in Table 2. Overall, TF decreased significantly (P<0.005) as did infection (*P* = 0.01). Seropositivity increased over the three years (*P*<0.001).

**Table 2:** Cross-sectional TF, infection, and seropositivity percentages of subjects who participated in each survey as shown in Fig 1.

Survey	TF (95% CI)	Infection (95% CI)	Seropositivity (95% CI)
Baseline	5.2 (4.4–6.2)	4.9 (4.1–5.9)	31.5 (29.7–33.4)
1^st^ Fup^a^	3.0 (2.3–3.8)	6.6 (5.6–7.7)	35.9 (33.9–38.0)
2^nd^ Fup	1.8 (1.3–2.5)	2.8 (2.2–3.7)	36.4 (34.4–38.5)
3^rd^ Fup	1.8 (1.3–2.5)	2.6 (2.0–3.4)	36.4 (34.3–38.5)

^a^ Follow-up

The age-specific seropositivity rates for each survey are shown in S1 Fig, where the increasing age of the cohort means that the youngest age groups with the lowest seropositivity rate are not represented at the three-year survey. S2 Fig shows the same age group at each survey point, suggesting the increase in seropositivity is driven by increases in the children aged five years and younger. The 9-year-olds at second and third follow-ups, who were 6-7-year-olds at baseline, had lower seropositivity rates than 9-year-olds at baseline (P = 0.047 and P = 0.0022, S2G Fig), likely because they lived through periods of lower overall TF and lower overall infection than the baseline group of 9-year-olds.

The cohort was divided into seven groups, based on distinct individual pattern of change in serostatus (Table 3). Of those who were seropositive at baseline, most children (n = 450, 86.2%) remained seropositive at all follow-ups; 63 (12.1%) were seroreverters. In a multivariable model adjusted for clustering (Table 4), seroreverters were younger (Odds Ratio [OR] = 0.88 for every one-year increase in baseline age, 95% CI = 0.79–0.99) and more likely to be female (OR = 1.77, 95% CI = 1.02–3.07). They also tended to live in a village with a baseline TF<5% (OR = 1.27, 95% CI = 0.68–2.39), although the result was not statistically significant. We did not have enough power to study the effect of infection rate on seroreversion as 96% of children lived in a village with zero infection at baseline. A test of interaction between age and sex was not significant. In sensitivity analyses including 96 participants lost to one follow-up who were either seronegative throughout or seroreverters, the results did not change.

**Table 3:** Characteristics of the cohort divided into each longitudinal serostatus group.

Groups	Serostatus at baseline and each follow-up +: seropositive; -: seronegative	n (%)	Average Age (years)	PercentFemale (%)	Average village TF at baseline (%)	Average village CT infection at baseline (%)
*Started seropositive (N = 522)*						
Group 1: seropositive throughout	+ + + +	450 (86.2^a^)	6.5	54.2	5.4	4.9
Group 2: seroreverters	+––– + +–– + + + –	63 (12.1^a^)	5.9	66.7	5.2	4.3
Group 3: transient change (only one seroreversion then seroconverted)	+–+ + + +–+	5 (1.0^a^)	6.0	20.0	4.6	4.1
*Started seronegative (N = 1197)*						
Group 4: seronegative throughout	––––	1011 (84.5^b^)	4.8	49.7	5.2	4.4
Group 5: seroconverters	– + + + ––+ + –––+	156 (13.0^b^)	4.3	57.1	5.3	5.1
Group 6: transient change (only one seroconversion then seroreverted)	––+– –+––	26 (2.2^b^)	4.8	53.8	5.3	4.7
Group 7: unstable	+–+– +––+ –+–+ –+ + –	8 (0.5^c^)	7.0	62.5	2.4	3.0

^a^ Denominator is the number of participants who started seropositive (N = 522)

^b^ Denominator is the number of participants who started seronegative (N = 1197)

^c^ Denominator is the number of all participants who showed up to all three follow-ups (N = 1719)

**Table 4:** Multivariable logistic regression analysis of factors associated with seroreversion or seroconversion in the longitudinal cohort.

	Unadjusted Odds Ratio (95% CI)	*P* value	Cluster Adjusted Odds Ratio (95% CI)	*P* value
*Factors for Seroreversion*				
Age	0.90 (0.80–1.00)	0.051	0.88 (0.79–0.99)	0.03
Sex^a^	1.69 (0.98–2.99)	0.065	1.77 (1.02–3.07)	0.04
Village TF <5% at baseline	1.22 (0.72–2.08)	0.47	1.27 (0.68–2.39)	0.46
*Factors for Seroconversion*				
Age	0.91 (0.85–0.98)	0.0087	0.92 (0.87–0.98)	0.005
Sex^a^	1.35 (0.96–1.90)	0.086	1.35 (0.94–1.95)	0.10
Village TF ≥5% at baseline	1.34 (0.95–1.88)	0.092	1.37 (0.78–2.39)	0.27

^a^ Reference is male.

Of those in the cohort who were seronegative at baseline, 1011 (84.5%) remained seronegative throughout the study; 156 (13.0%) were seroconverters. In a multivariable model adjusting for clustering (Table 4), seroconverters were younger (OR = 0.92 for every one-year increase in baseline age, 95% CI = 0.87–0.98). They also tended to be female (OR = 1.35, 95% CI = 0.94–1.95) and more likely to live in a village with a baseline TF≥5% (OR = 1.37, 95% CI = 0.78–2.39), although the latter two results were non-significant. In sensitivity analyses including the 203 participants lost to one follow-up who were either seronegative throughout or seroconverters, female sex was significantly associated with seroconversion (OR = 1.51, 95% CI = 1.08–2.11). The odds of seroconverters residing in a village with estimated TF ≥5% increased to 1.52 but was not statistically significant (*P* = 0.13). Overall, younger children and female children had more fluidity in serostatus, and residence in a village with TF≥5% likely increases the risk of seroconversion.

**Fig 2:**
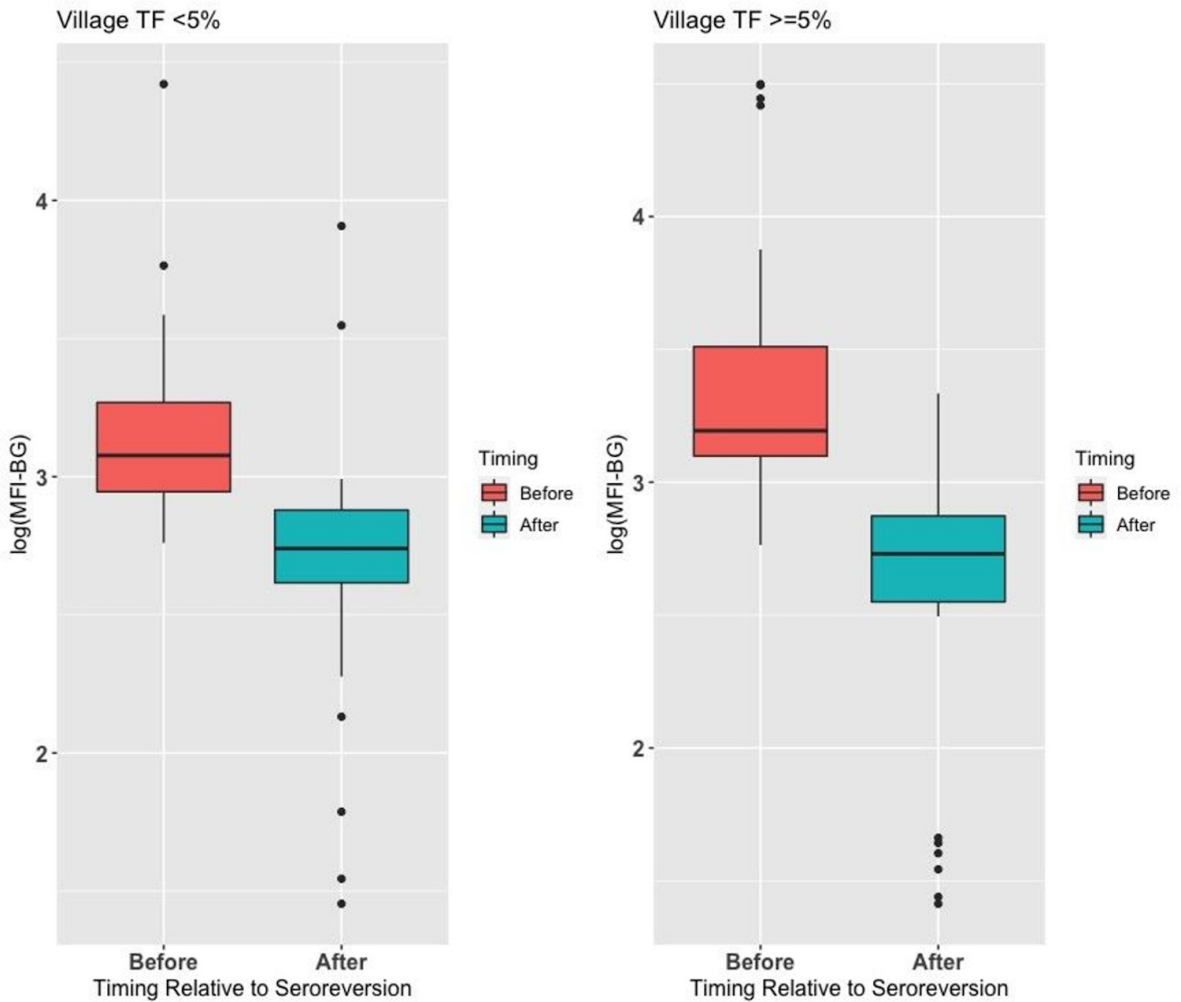
Log of MFI-BG in seroreverters in the survey immediately before and in the survey that defined seroreversion.

We examined the MFI-BG in seroreverters and the effect of village TF prevalence at baseline on the change in intensity of antibody response. As shown in Fig 2, the change in log MFI-BG was similar in children in both sets of villages. We evaluated the significance in children whose pre- and post- seroreversion survey used the same bead sets, and the mean decrease in log MFI-BG was 0.54 (*P*<0.001).

**Fig 3:**
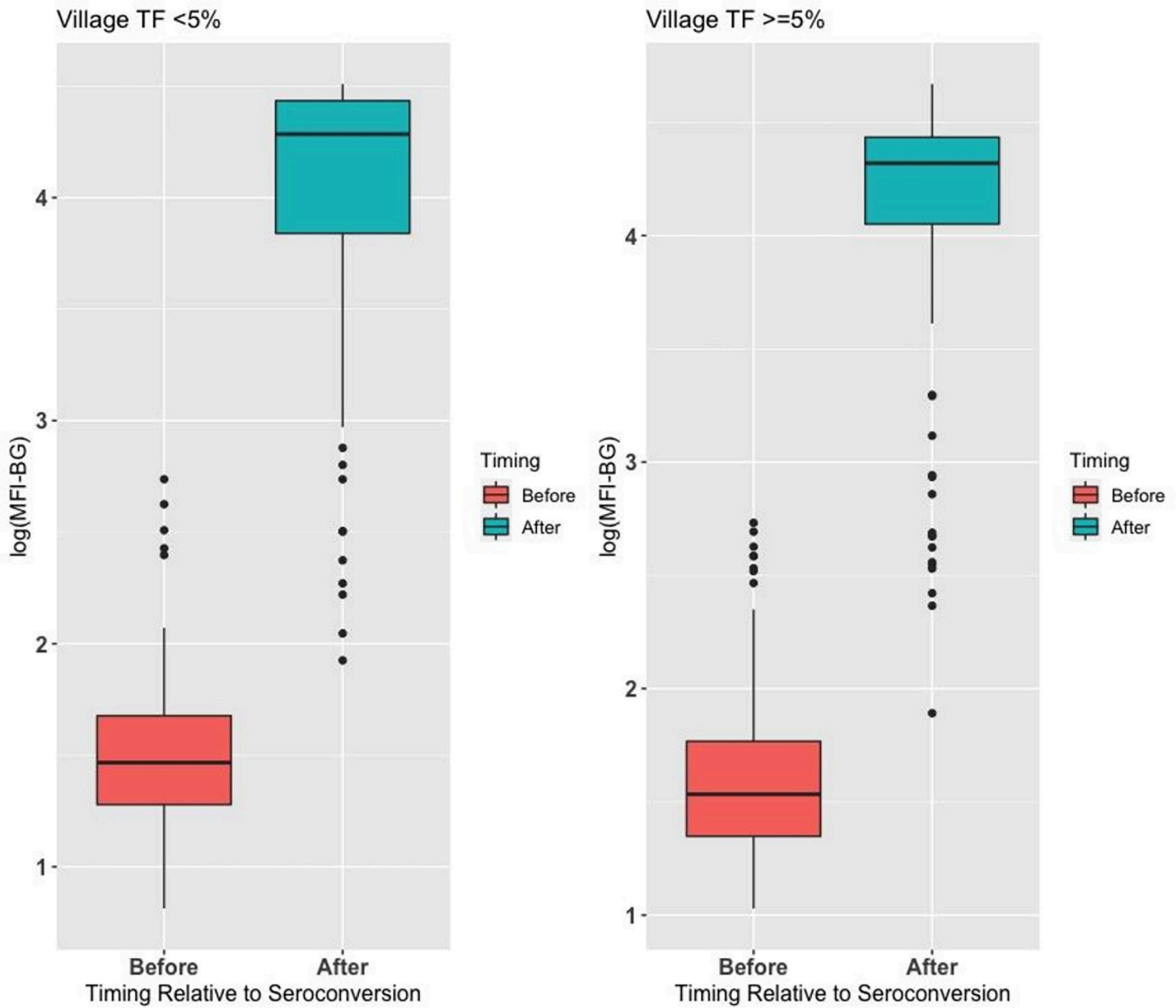
Log of MFI-BG in seroconverters in the survey immediately before and the survey that defined seroconversion.

The change in MFI-BG was similarly evaluated among children who were seroconverters (Fig 3). There was no effect on the intensity of change according to estimated village TF prevalence at baseline. We evaluated the significance in children whose pre- and post- seroconversion survey used the same bead sets, and the mean increase in log MFI-BG was 2.49 (*P*<0.001).

There were 31 (2%) children who experienced a single or transient seroconversion or seroreversion (Groups 3 and 6). We examined the effect of MFI-BG values falling in the indeterminate range on these findings. Only one child (3% of the 31) had the single change with a value of MFI-BG in the indeterminate range. The other children in this group did not have MFI-BG values in the indeterminate ranges. The data suggests that these transient changes in serostatus are real and not due to values in the indeterminate ranges. Twenty of the 26 children (76.9%) who had one transient seroconversion were under age 6.

Of the longitudinal cohort, only eight participants (0.5%) had unstable serostatus (Group 7), frequently changing between seropositive and seronegative. The apparent instability of their serostatus can largely be explained by MFI-BG values in the indeterminant ranges, which affected five of the eight children (Table 5). Notably, all children in the unstable group were over five years old and lived in villages with TF≤5%. No one in the unstable group had TF/TI or Ct infection.

**Table 5:** MFI-BG (log MFI–BG)^a^ of the unstable serostatus group at baseline and follow-ups. Highlighted cells contain MFI-BG values that fell in the indeterminant ranges.

Participants	Baseline	1^st^ follow-up	2^nd^ follow-up	3^rd^ follow-up
*Serostatus*	+	–	+	–
Participant 1	690 (2.84)	449 (2.65)	1318 (3.12)	128 (2.11)
Participant 2	983 (2.99)	769 (2.89)	1187 (3.07)	521 (2.72)
Participant 3	1554 (3.19)	856 (2.93)	1321 (3.12)	357 (2.55)
*Serostatus*	+	–	–	+
Participant 4	673 (2.83)	589 (2.77)	657 (2.82)	1017 (3.01)
*Serostatus*	–	+	–	+
Participant 5	270 (2.43)	1583 (3.20)	607 (2.78)	1928 (3.29)
*Serostatus*	–	+	+	–
Participant 6	30 (1.48)	8490 (3.93)	2374 (3.38)	425 (2.63)
Participant 7	105 (2.02)	1619 (3.21)	1006 (3.00)	583 (2.77)
Participant 8	324 (2.51)	1710 (3.23)	1015 (3.01)	506 (2.70)

^a^ Cut-offs for positivity: 575 (log MFI-BG = 2.76) at baseline, with an indeterminate range of 466–683, and 989 (log MFI-BG = 2.99) at follow-ups, with an indeterminate range of 791–1187.

## Discussion

In our cohort of children in a formerly trachoma hyperendemic district, we found that serostatus was largely stable, with approximately 85% of children retaining baseline serostatus over the three-year period. This was not 100%, as observed 6 months following MDA in a hyperendemic community [7], but we followed our cohort for a longer period, in a low endemic district but with some communities having TF prevalence estimated above and below 5%. Although one might expect to see more seroreversion after MDA, the two rounds of MDA in our study period did not, for the most part, seem to affect the overall stability of serostatus in children. This is consistent with our short-term results that showed a modest impact of MDA on MFI-BG, and a slight but not striking increase in seroreversion rates after MDA [13]. Less than 1% of children had truly unstable serostatus, largely due to MFI-BG values in the indeterminate range. Stability is a desirable quality when estimating seroconversion rates from age specific prevalence, if the assumption is made that once antibodies are acquired, they are not lost. However, such was not the case in our study, as we found strong evidence for seroreversion.

Seroconversion and seroreversion occurred at rates of 13% and 12% respectively during the three-year period. Very few children had MFI-BGs in the indeterminate ranges around the cut-offs, so the observed seroreversion and seroconversion are not artifacts from indeterminate ranges. The previous study in a hyperendemic community reported no seroreversion following MDA but was likely related to the short follow-up period and the high level of infection prior to MDA [7]. Previous modeling estimated the half-life of seroreversion to be 26 years [18], while our study suggested shorter timelines.

Our observation of seroreversion, and what seems to be real transient seroconversions, makes biological sense. While it is unclear how many infections are needed to maintain seropositivity, a single infection is not enough [19]. The higher cumulative antigen load over time boosts memory B cells and long-lived plasma cells that secrete high numbers of circulating antibodies [20], which then help to maintain seropositivity, even in the absence of continued disease exposure. Therefore, it is not surprising that we found younger children to have more fluidity in serostatus than older children. Older children have had more episodes of Ct exposure in their lifetime, so they are more likely to develop long-lasting immune memory and stable serostatus. This is also demonstrated in the group with transient changes in serostatus, most of whom were under age six. Our data support a recommendation that modeling efforts to estimate seroconversion should also account for age specific estimates of seroreversion in order not to underestimate seroconversion. There might be concern that seropositivity in the youngest children is due to transfer of maternal antibodies to urogenital Ct strains and that the waning of maternal antibodies contributed to the loss of seropositivity. However, the decline of maternal antibodies occurs between 6–12 months [21], which should only contribute to seroreversion in children <1 year old. No child in our study was under age one.

Seroconversion was also observed in this cohort, despite residing in a district where estimated trachoma and infection rates were low. Living in villages where the estimated TF rate based on our sample was ≥5% was associated with greater odds of seroconversion but it was not statistically significant. The intensity of the seroconversion did not differ by whether the estimated TF was less than 5% compared to 5% or more. As with seroreversion, the risk of seroconversion was significantly higher in the younger children. The greater risk of seroconversion in younger children likely reflects two factors. First, younger children are less likely to be seropositive, so as a group when they encounter an infection, there are more of them eligible to seroconvert. Second, in general, younger children have more infection and trachoma than older children suggesting their risk of exposure is greater than that in older children.

Monitoring or surveillance for trachoma should be for the purpose of detecting resurgence or re-emergent disease over a specific time frame. Our data suggest that the younger age groups, specifically 5 years and under, were better suited as the sample to reflect the greatest risk of seroconversion from repeated exposure to infection compared to the older age groups. In another study, following a district impact survey which found TF prevalence to be <5%, a subsequent survey found that the age group born after this impact assessment provided the serologic evidence that re-emergence had occurred [11].

We found that female children were more likely to seroconvert, and this association was statistically significant in sensitivity analyses. One possible reason is that young girls often assist in looking after younger siblings, which puts them in constant contact with other children. As young children are the main reservoir for Ct, female children are at higher risks of Ct infection than their male counterparts [22]. The levels of infection in this study were so low that further exploring risk factors in the girls was not possible.

Female children were also more likely to serorevert, suggesting more instability in their serostatus. While adult females tend to show greater antibody responses than males, the sex-based variation in immune responses can be more ambiguous in childhood before sex steroids start to exert major effects [23]. In fact, B cell numbers are comparable in male and female children [24,25]. In a study on the serum immunoglobulin levels in Nigeria, female children aged 5–12 were found to have equivalent IgG levels as male children, while female adults had significantly higher values of IgG compared to male adults [26]. The relative fluidity in female children’s response to trachoma antigen could be due to some sex difference in immunity that is yet unknown. To our knowledge, there is scarce research on the sex difference in serology in children, especially in those under 10 years old. More studies in this area are needed.

Only eight out of the 1719 children were in the unstable group, largely explained by MFI-BG values falling in the indeterminate range. If the serostatus instances where the values fall in the indeterminate range are instead counted as the alternate, then these children fall into a more regular pattern. Participants 2 and 3 in this group would be seroreverters, and participants 7 and 8 would have transient seroconversions. Only three children were truly alternating between seropositivity and seronegativity.

There are limitations to the study. First, the data reflect a formerly hyperendemic district, and the results might not apply in districts that have eliminated trachoma a long time ago, where seropositivity even in older ages may be low. However, the low TF and low infection in the district do reflect the condition expected during surveillance for trachoma re-emergence, where serology would be of most use. Second, two rounds of MDA within a short period occurred during the study period. Without MDAs, there would likely have been more seroconversions. Additionally, about 70% of children showed up to all follow-ups. Lost to follow up may theoretically lead to biases if there is differential inclusion of those more likely to seroconvert or serorevert. There were more baseline seropositive children in those who were lost to two or three follow-ups. However, there was no age or sex difference between seropositives who were lost to follow-ups and seropositives who remained in the study, so they were not more or less prone to seroreversion. Our sensitivity analyses using those missing only one follow-up did not affect any of our findings, except to add more children and make some associations statistically significant. Another limitation is the use of two different bead sets. We have previously reported the reproducibility of serostatus determination using different bead sets with different cut-off values for seropositivity, suggesting the use of two bead sets had limited impact on our findings [27].

In summary, our study in a cohort of children demonstrated the dynamics of seroconversion and seroreversion in this formerly endemic district with overall low rates of infection and disease. The finding of overall long-term stability in serostatus over time provides reassurance that cross-sectional studies likely reflect seropositivity rates over at least a three-year period. However, there is a dynamic interplay of seroconversion and seroreversion, especially in young children. Modeling efforts in serology in children should account for seroreversion to avoid underestimating the seroconversion rate. In addition, the dynamic nature of change in serostatus is most pronounced in the youngest children, so for monitoring purposes, programs should consider using the youngest age group as the target population. Our data suggest using the age group 5 years and younger, but the age group may be tailored to the specific epidemiology of the district and other factors, such as time since MDA cessation.

## Supporting information

S1 Data Excel spreadsheet with raw data. (XLSX)

S1 Fig Age-specific seropositivity rate at baseline (A) and each follow-up visit (B, C, and D).
(DOCX)

S2 Fig Seropositivity rate for the same age group at each survey point, for ages 3–9 years. Statistically significant differences from baseline are starred.
(DOCX)
