# Supplementary figures and images for: Risk of seroconversion and seroreversion of antibodies to Chlamydia trachomatis pgp3 in a longitudinal cohort of children in a low trachoma prevalence district in Tanzania
Source: PLoS Negl Trop Dis. 2022 Jul 13;16(7):e0010629. doi: 10.1371/journal.pntd.0010629 (PMC9312410; doi:10.1371/journal.pntd.0010629)

A B

**
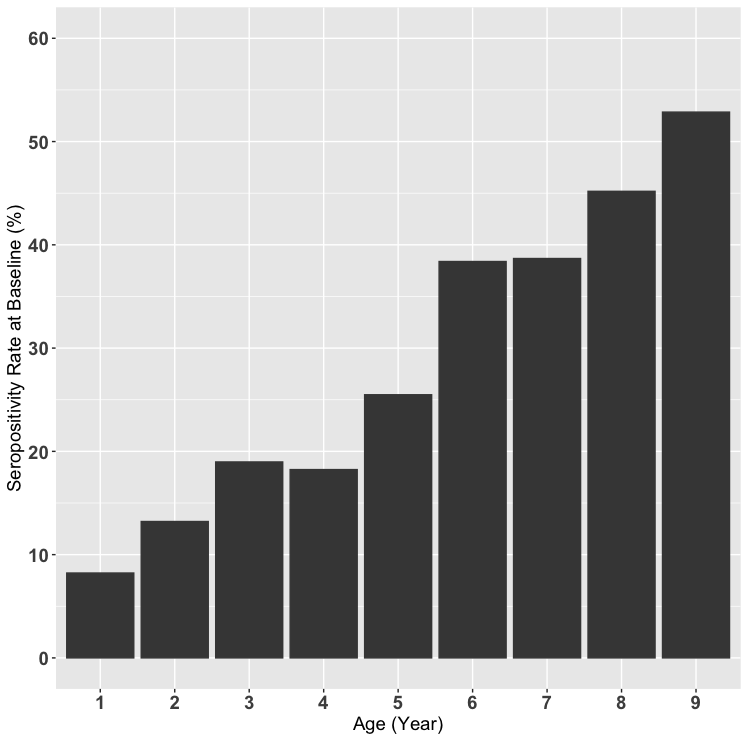

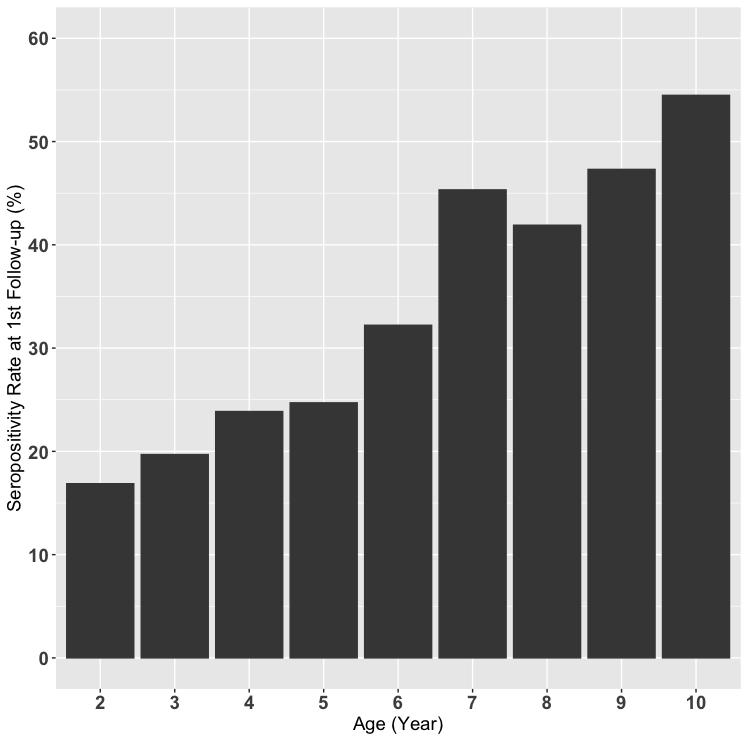
**

C D

**
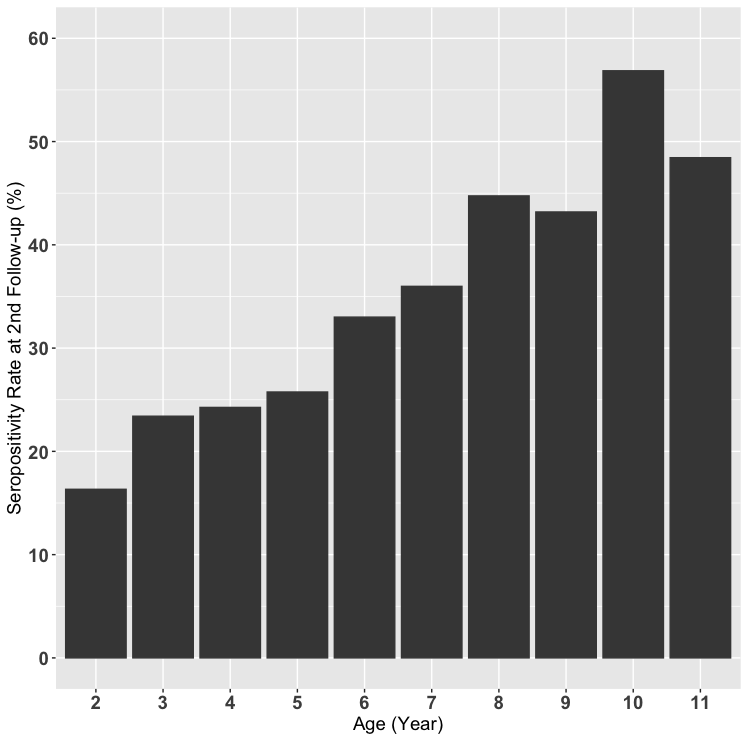

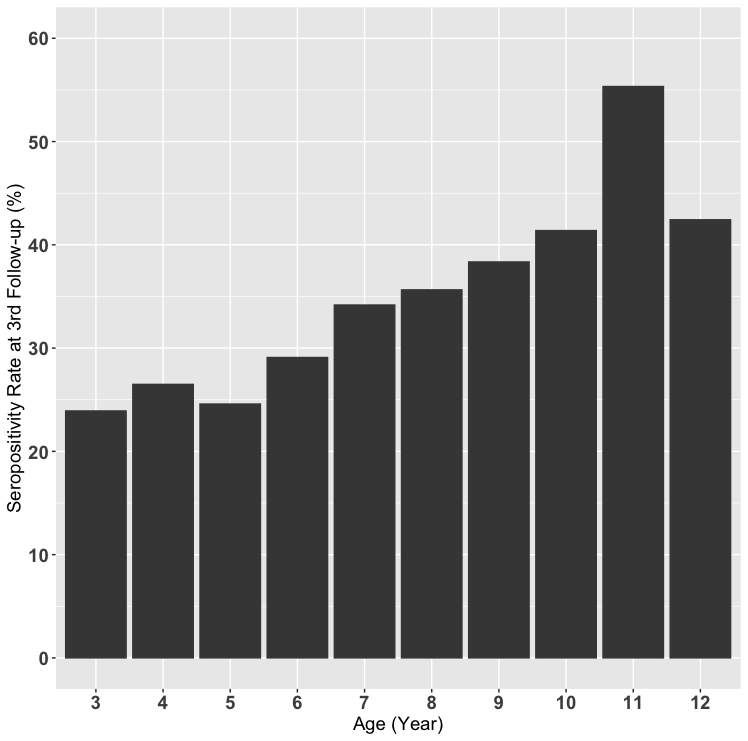
S1 Fig.** Age-specific seropositivity rate at baseline (A) and each follow-up visit (B, C, and D).

Supplement: S1 Fig — Age-specific seropositivity rate at baseline (A) and each follow-up visit (B, C, and D). (DOCX) [file pntd.0010629.s002.docx]
