# Supplementary material for: Risk of seroconversion and seroreversion of antibodies to Chlamydia trachomatis pgp3 in a longitudinal cohort of children in a low trachoma prevalence district in Tanzania
Source: PLoS Negl Trop Dis. 2022 Jul 13;16(7):e0010629. doi: 10.1371/journal.pntd.0010629 (PMC9312410; doi:10.1371/journal.pntd.0010629)
Supplement: S2 Fig — Statistically significant differences from baseline are starred. (DOCX) [file pntd.0010629.s003.docx]

A B C


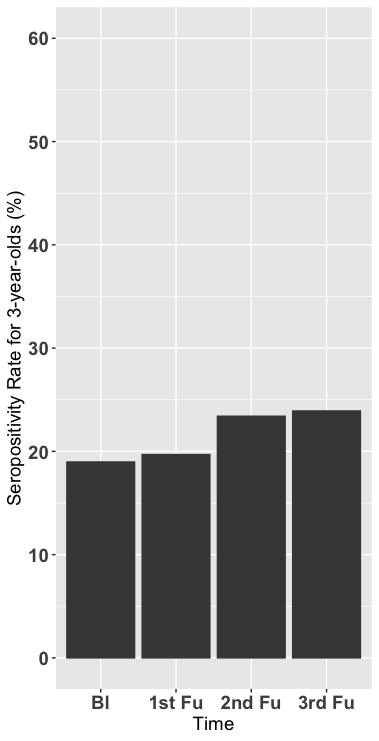

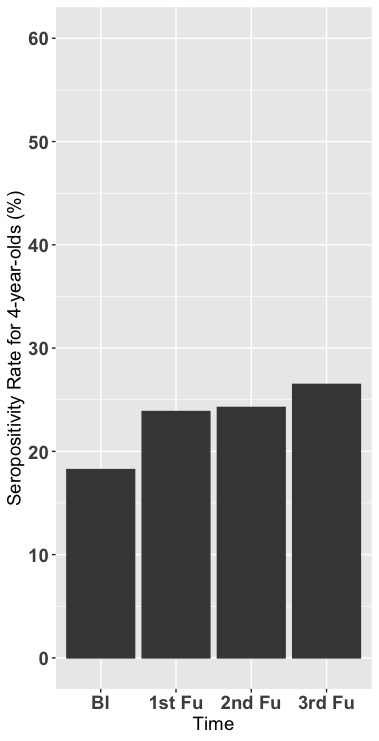

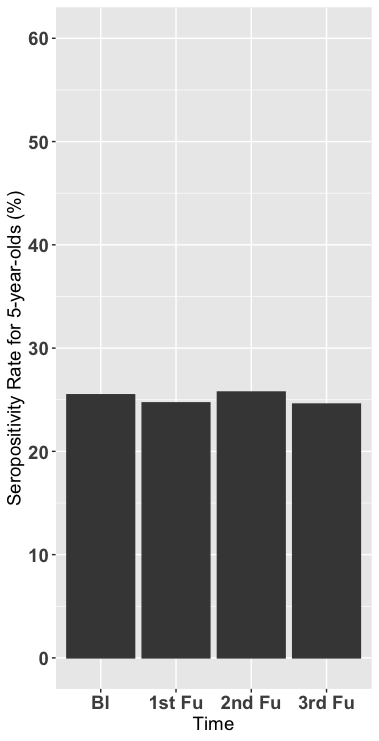


D E F


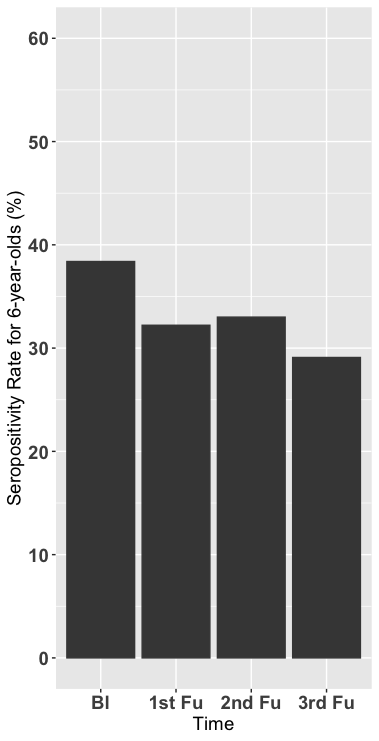

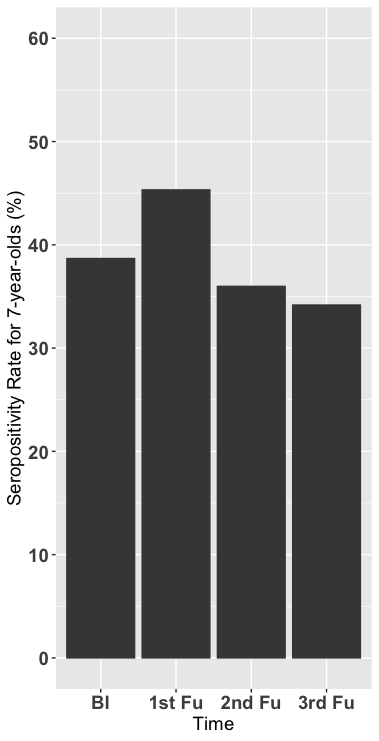

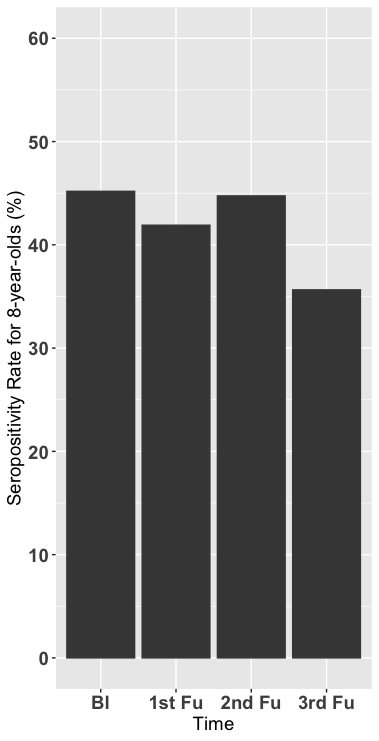


G


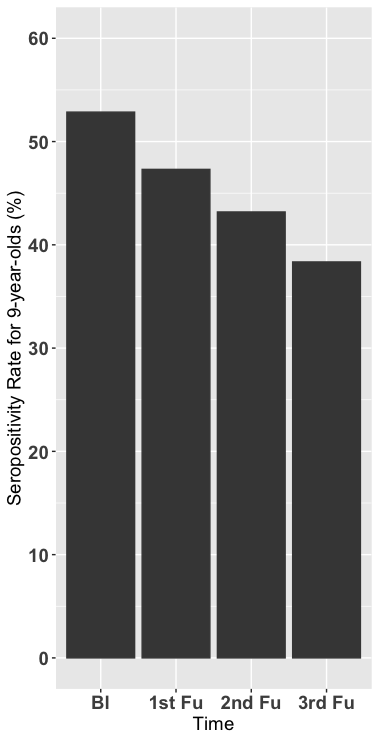


**S2 Fig.** Seropositivity rate for the same age group at each survey point, for ages 3-9 years. Statistically significant differences from baseline are starred.
